# Burden and trend of cardiovascular diseases in youths aged 0–19 years in China, Asia, and the world, with forecasts to 2036: a systematic analysis of the global burden of disease study 2021

**DOI:** 10.3389/fpubh.2025.1653981

**Published:** 2026-01-23

**Authors:** Jin Yu, Shanshan Zhu, Zeping Wang, Yongyao Shen, Sheng Ji, Yongjin Guo, Liying Jiang

**Affiliations:** 1 Graduate School, Shanghai University of Traditional Chinese Medicine, Shanghai, China; 2 College of Public Health, Shanghai University of Medicine & Health Sciences, Shanghai, China; 3 Department of Human Resources, Jiangyan District Center for Disease Control and Prevention, Taizhou, Jiangsu, China; 4 Department of Human Resources, Jiangyan District Health Commission, Taizhou, Jiangsu, China; 5 School of Nursing and Health Management, Shanghai University of Medicine & Health Sciences, Shanghai, China; 6 Jiading Central Hospital, Shanghai University of Medicine & Health Sciences, Shanghai, China

**Keywords:** cardiovascular diseases, children and adolescents, disease burden, world, Asia, China

## Abstract

**Objective:**

To combat the harmful impacts of cardiovascular diseases (CVDs) on public health, it is crucial to understand the epidemiologic features and disease burden. The study aims to identify the trends of CVDs burden in youths aged 0–19 years from 1990 to 2021 across the global, Asian, and Chinese contexts to inform more effective strategies and actions.

**Methods:**

The data from the Global Burden of Diseases and Risk Factors Study (GBD) 2021 were analyzed, stratified by sex, age, the socio-demographic index (SDI) regions, disease subtypes, respectively. We also employed the Auto-Regressive Integrated Moving Average (ARIMA) to predict the future burden of CVDs up to 2036.

**Results:**

In 2021, DALY rate of CVDs among children and adolescents were 303.140 (268.480–343.800) globally, 268.730 (242.730–298.350) in Asia and 148.800 (126.510–173.620) in China. It exhibited significant declined trends from 1990 to 2021, with average annual percentage changes (AAPCs) of −2.447, −2.665, and −4.379, respectively (all *p* < 0.001). Compared with males, females had relatively higher prevalence of CVDs globally (797.060 vs. 791.140 per 100,000). Rheumatic heart disease served as the most prominent subtype across the world. Non-optimal temperatures emerged as the primary risk factor for CVDs-related disability-adjusted life years (DALYs). The CVDs incidence rate is predicted to rise to 108.861 per 100,000 globally, while the rate in Asia remains steady and decreases in China in 2036.

**Conclusion:**

A substantial global burden of CVDs in youths aged 0–19 years remains the pressing public health issue in 2021. The burden of overall and type-specific CVDs varies by age, sex, SDI, regions and countries. Current and future challenges in CVDs prevention for youngsters implied by the epidemiologic features are highlighted in this study.

## 1 Introduction

Cardiovascular diseases (CVDs) are the leading cause of morbidity and mortality, inflicting substantial social and economic costs (1, 2). According to the Global Burden of Disease (GBD) 2021 study and the World Heart Report 2023, CVDs are responsible for 20.5 million deaths globally, accounting for approximately one-third of total deaths (3, 4). The Asia exhibits tremendous disparities and adversities in wealth status, rendering it a critical focal point for CVDs prevention and control efforts. According to the China Cardiovascular Health and Disease Report 2023, CVDs serve as the leading cause of death, accounting for 48.980% of deaths in rural areas and 47.350% in urban regions in 2021 (5, 6).

CVDs mainly appear in the middle-age adults and older adults. CVDs in adults encompass a range of disorders affecting the blood vessels, including coronary artery disease (CAD), cerebrovascular disease (CeVD), and peripheral vascular disease (PVD). However, some CVDs tend to be triggered in younger population in recent decades. For example, the prevalence of hypertension in youths in China was 13.000% in 2019, 13.200% in girls (12.700% in boys), also 14.100% in the rural (11.900% in the urban), representing an upward trend with age (7).

As a major cause of CVDs mortality in youths in developing countries, rheumatic heart disease can cause approximately 250,000 deaths every year and reaches a peak between the 20–29 age. DALYs for alcoholic cardiomyopathy rises rapidly from the age of 25 (8). Moreover, infective endocarditis in youths without congenital heart disease was really similar to that in adults and caused short-term deaths, combined with acute heart failure, compared with those with congenital heart disease (9, 10).

CVDs in youths present chronic and complex conditions inflicted by biological contexts and environmental factors (7). Adolescence is an important period for physical and mental development being more neglected for health coverage (11, 12). Previous studies suggested that CVDs in youths presented a substantial share in mortality burden with that of adults (13). Atherosclerosis can begin early in lifetime and develop undetected for an extended time before going into an advanced phase with clinically presentable manifestations (14, 15). The underlying mechanisms and features of pediatric CVDs exhibit specific patterns and differ commonly compared with adults (6, 16). Most importantly, the pathological changes from childhood to adulthood can predict fatal or nonfatal CVD in later middle lifetime.

Obesity, high blood pressure, and abnormal plasma lipids are well-established CVDs risk factors appearing in childhood and adolescents (17). If these risk factors, being inherited or acquired, continue to be unmodified, high-risk conditions are prone to develop the adverse outcomes when approaching adults. Previous research focuses on clinical factors such as obesity, blood pressure, and cholesterol that align with CVDs in adults, while researches on pediatric CVDs tend to focus on lifestyle-related factor, such as dietary quality, physical activity and psychological characteristics (18). Critical barriers to early prevention are the difficulty in accurately evaluating CVDs risk in youths due to the subclinical nature of CVDs. Evidence on potential risk factors for CVDs among young population globally is limited. These gaps might be involved in informing effective strategies and prevention efforts to reduce CVDs burden in young population worldwide.

There is an urgent need to develop and implement effective and targeted strategies for the primary prevention of CVDs. Understanding the temporal trends in the epidemiological trends of overall CVDs in youths and its attributable risk factors is crucial worldwide. To the best of our knowledge, the features of CVDs epidemics in the young population across the world have yet to be presented, also a comprehensive profile in Asia and China. Therefore, we aimed to report CVDs epidemics and burdens and how they changed from 1990 to 2021 using newly updated estimates of total and specific CVDs in young population aged 0–19 at global, regional, and national levels, stratified by age, sex, regions, countries, and sociodemographic index (SDI), also along with predictions to the 2036 year.

## 2 Materials and methods

### 2.1 Study data

The data were obtained from the Global Burden of Disease (GBD) 2021 database. To ensure robustness, the GBD study employs DisMod-MR and Spatiotemporal Gaussian Process Regression to address data gaps and missing data and applies standardized case definitions and inclusion criteria for variable selection. The detailed methods for the GBD have been described elsewhere (3, 19). The parameters, including fatal metrics (incidence rates, prevalence estimates, and YLLs), as well as non-fatal metrics (DALYs, mortality, and YLDs) were extracted from the GBD 2021 repository^1^ published by the Institute for Health Metrics and Evaluation, which synthesizes data by sex, age and SDI, from 204 countries through 12,765 data sources (19, 20). Ethical approval and informed consent were waived because the GBD is publicly available and no identifiable information was included in the study. The study incorporated projected trajectories of these metrics to 2036 employing the modeling frameworks to forecast temporal trends.

YLL quantifies premature mortality by calculating the difference between age at death and the standard life expectancy, using demographic-specific reference standards. YLD measures non-fatal health loss through disability weight (DW) assignments (0 = full health, 1 = death). As the principal composite metric in burden of disease studies, DALY integrates mortality and morbidity through: DALY = YLL + YLD.

SDI is a composite measure of a country’s lag distributed income per capita, average years of schooling, and the fertility rate in females younger than 25 years. This study categorized countries into five SDI levels to explore the correlation between burden and socioeconomic development.

### 2.2 Definitions of CVDs in children and adolescents

Subtypes included hypertensive heart disease, ischemic disease, non-rheumatic valvular heart disease, rheumatic heart disease, stroke, aortic aneurysm, cardiomyopathy and myocarditis, endocarditis, and other cardiovascular and circulatory diseases. Age-specific burden of CVDs was being categorized into four age groups: ≤5 years, 5–9 years, 10–14 years, and 15–19 years.

This study focused on the potential risk factors associated with CVDs in children and adolescents under 20 years old, as identified in the database. All causes were analyzed via the GBD hierarchical framework to identify predominant contributors to health loss. We categorized diseases into three level groups: (1) communicable, maternal, neonatal, and nutritional diseases; (2) non-communicable diseases (NCDs); and (3) injuries. For granular analysis of chronic disease patterns, we exclusively focused on level 2 and level 3 causes within the NCDs category (21). The definitions of all risk factor were abstracted from the Global Health Data Exchange (GHDx) platform.

### 2.3 Statistical analyses

Descriptive analyses were used to characterize the burden of CVDs separated by gender, age group, disease subtypes at three hierarchical levels: global, Asia, and China. The trends across 1990–2021 were evaluated using average annual percent change (AAPC). The future trends (2021–2036) were predicted using the ARIMA calculated by the Joinpoint Regression Program (Version 5.4.0) and R studio (Version V4.4.1) (22). The predictive validity of the ARIMA model was secured by achieving stationarity and optimizing its core parameters, including the autoregressive (*p*), differencing (*d*), and moving-average (*q*), respectively, via Akaike information criterion value, followed by rigorous diagnostic checks for residual white noise tests and a battery of forecast error measures. All uncertainty intervals were generated through 1,000 posterior draws from ARIMA, with final estimates representing the 2.5th–97.5th percentile ranges. Statistical significance was defined as non-overlapping 95%UIs for *p* < 0.05 in regression models.

## 3 Results

In 2021, the number of CVDs among children and adolescents were 20.928 million (95%CI: 16.652–25.860) globally, 8.862 million (95%CI: 7.018–10.910) in Asia, and 2.131 million (95%CI: 1.697–2.667) in China, with prevalence rate of 794.020 (95%CI: 631.780–981.130), 614.030 (95%CI: 486.320–755.900), 637.670 (95%CI: 507.670–797.820) per 100,000 population, respectively. While metrics showed downward trends (all AAPC <0), excepted for four exceptions: global incidence (0.314, 95%CI: 0.271–0.357), global prevalence (0.506, 95%CI: 0.439–0.574), global YLDs (0.203, 95%CI: 0.172–0.234), and Asian prevalence (0.163, 95%CI: 0.080–0.245) (Table 1).

**Table 1:** Absolute numbers, rates and annual percentage changes in the incidence, prevalence, death, DALYs, YLLs and YLDs of CVDs in China, Asia and the world from 1990 to 2021 (rate: per 100,000 people).

Measures	Metrics	Global		Asian		China	
		1990	2021	1990	2021	1990	2021
Incidence (95%UI)	Number	2134448.334 (1667532.634, 2715350.961)	2738540.036 (2074960.889, 3589001.341)	1260791.653 (983610.881, 1610107.923)	1245604.688 (949258.181, 1631122.914)	542194.445 (426129.901, 692967.785)	311749.978 (241405.915, 394797.853)
	Rate	94.565 (73.834, 120.223)	103.966 (78.722, 136.168)	90.873 (70.895, 116.051)	86.304 (65.771, 113.015)	121.830 (95.750, 155.708)	93.252 (72.219, 118.168)
	AAPC	0.314 (0.271, 0.357)*		−0.158 (−0.245, −0.072)*		−0.866 (−0.963, −0.769)*	
Prevalence (95%UI)	Number	15345812.633 (12390966.030, 18569761.065)	20928983.821 (16652804.846, 25860941.238)	8122755.943 (6502245.077, 9980430.563)	8862193.531 (7018989.524, 10910163.870)	3389825.062 (2658247.591, 4252937.392)	2131751.776 (1697153.960, 2667155.732)
	Rate	679.451 (548.623, 822.192)	794.028 (631.785, 981.137)	585.462 (468.661, 719.357)	614.034 (486.324, 755.932)	761.687 (597.303, 955.626)	637.673 (507.679, 797.827)
	AAPC	0.506 (0.439, 0.574)*		0.163 (0.080, 0.245)*		−0.554 (−0.666, −0.441)*	
DALYs(95%UI)	Number	14791657.733 (13368075.392, 16966494.143)	7990383.383 (7076648.615, 9061976.187)	8493415.375 (7649410.780, 9723420.846)	3878556.524 (3503271.467, 4305985.699)	2625984.855 (2335722.414, 2924035.846)	497456.256 (422933.349, 580424.943)
	Rate	654.912 (591.883, 751.249)	303.148 (268.489, 343.806)	612.18 (551.354, 700.835)	268.736 (242.734, 298.353)	590.055 (524.838, 657.039)	148.8 (126.518, 173.622)
	AAPC	−2.447 (−2.496, −2.399)*		−2.665 (−2.737, −2.592)*		−4.379 (−4.512, −4.246)*	
YLDs (95%UI)	Number	1133510.583 (795222.227, 1557997.379)	1407909.419 (963907.123, 1962293.482)	642339.183 (455903.992, 879745.772)	644440.810 (451162.786, 886073.245)	271284.828 (189858.458, 375527.229)	160950.488 (112157.612, 222007.532)
	Rate	50.190 (35.211, 68.982)	53.419 (36.572, 74.459)	46.3 (32.863, 63.419)	44.655 (31.263, 61.394)	60.964 (42.669, 84.383)	48.143 (33.556, 66.414)
	AAPC	0.203 (0.172, 0.234)*		−0.115 (−0.180, −0.050)*		−0.762 (−0.826, −0.698)*	
Deaths (95%UI)	Number	163751.281(147009.56, 190128.867)	81419.723(71596.254, 91661.148)	94993.86(84680.12, 109562.09)	40649.273(36507.624, 45073.963)	28503.035(25206.187, 32175.626)	4271.156(3611.389, 4919.947)
	Rate	7.253(6.519, 8.428)	3.098(2.726, 3.488)	6.852(6.124, 7.923)	2.824(2.535, 3.162)	6.409(5.66389, 7.232)	1.284(1.085, 1.473)
	AAPC	−2.627 (−2.712, −2.541)*		−2.840 (−2.926, −2.754)*		−3.921 (−4.048, −3.793)*	
YLLs (95%UI)	Number	13658147.150 (122280479.82, 15924522.575)	6582473.965 (5754595.45, 7443538.696)	7851076.195 (6979047.653, 9091512.534)	3234115.776 (2896610.96, 3611770.84)	2354700.039 (2080801.974, 2666536.078)	336505.776 (285264.269, 388332.137)
	Rate	604.723 (541.412, 705.071)	249.73 (218.32, 282.490)	565.882 (503.034, 655.298)	224.085 (200.776, 250.250)	529.165 (467.557, 599.178)	100.669 (85.330, 116.169)
	AAPC	−2.753 (−2.896, −2.611)*		−2.996* (−3.081, −2.910)		−3.971 (−4.101, −3.841)*	

*p<0.001.

**Figure 1:**
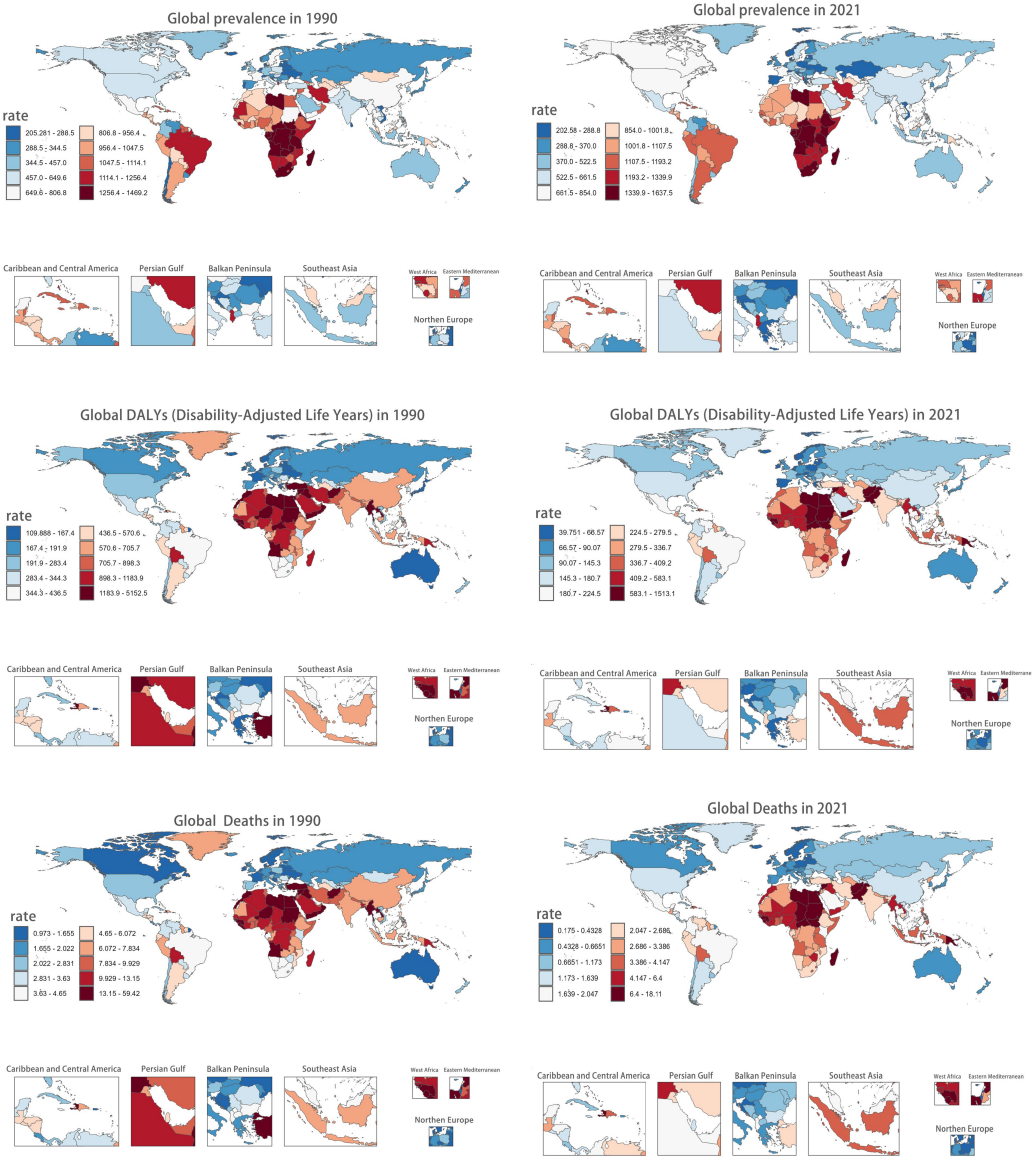
Burden of CVDs across 204 countries/territories among children and adolescents, in 1990 and 2021.

Figure 1 mapped the global CVDs burden for children and adolescents synthetically. Briefly, DALY rates were predominantly concentrated in African in both 1990 and 2021. In 2021, the three highest DALY rates were in Republic of Niue: 1513.120 (95%CI: 1336.220–1721.500); Tokelau: 1371.700 (95%CI: 1055.120–1624.180); State of Libya: 1264.530 (95%CI: 986.530–1600.860) per 100,000 population, respectively. Conversely, the lowest DALY rates predominantly clustered in high-SDI nations: Republic of Cyprus: 42.600 (95%CI: 35.300–51.420); State of Israel: 42.610 (95%CI: 36.040–51.050); Swiss Confederation:45.090 (95%CI: 38.350–53.070). A strong inverse correlation linking higher SDI with lower DALYs rates existed (Supplementary Figures 1, 2).

### 3.1 Age-specific CVDs metrics in children and adolescents

**Figure 2:**
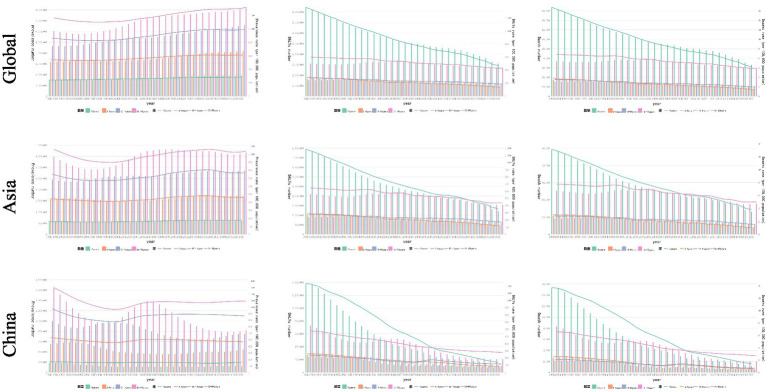
Temporal trends in prevalence, DALYs, and mortality rate of CVDs in children and adolescents by age in China, the Asia, and the world, from 1990 to 2019.

Globally, CVDs burden among children and adolescents exhibited a clear age-dependent divergence between fatal and non-fatal outcomes (Supplementary Table 1). With age advancing, DALYs and mortality gradually declined, while the prevalence and incidence showed gradual increase slowly. These trends were roughly same in China, Asia and the world (Figure 2).

From 1990 to 2021, age-specific disparities revealed the distinct patterns as follows: for children<5 years, most metrics such as the Chinese DALY rate (1236.893–140.450) exhibited uniform declines, comparing with the rising metric of global and Asian prevalence/YLDs rate (global prevalence 242.532–287.023); for those 5–9 years: global incidence, prevalence, YLDs and Asian prevalence rate (89.082–96.250, 543.190–611.981, 41.613–42.840, 444.327–459.865 respectively) all increased, whereas other metrics declined significantly; for 10–14 years children: the rate of global incidence, prevalence, YLDs and Asian prevalence (109.256–122.716, 864.346–989.620, 62.032–64.990, 1062.133–767.640, respectively per 100,000 population) have shown an increasing trend, whereas the other Asian and Chinese metrics exhibited stable; among 15–19 years children, only global incidence (124.814–140.525), prevalence (1163.296–1320.248), and YLDs (83.510–86.055) continued to rise through the period (Supplementary Table 1).

Notably, DALYs, mortality, and YLLs demonstrated a unique age hierarchy: the highest value [−3.412 (95%CI: −3.301 to −3.524); −3.526 (95%CI: −3.425 to −3.626); −3.522 (95%CI: −3.422 to −3.622)] existed in children under 5 years, followed by 15–19, 10–14, and 5–9 years at the global level. In Asia and China, accelerated reductions in mortality aged 5–9 years repositioned this specific group from the highest to the second-highest by 2021 (Supplementary Table 1).

### 3.2 Sex-specific CVDs and SDI-specific CVDs metrics in children and adolescents

**Figure 3:**
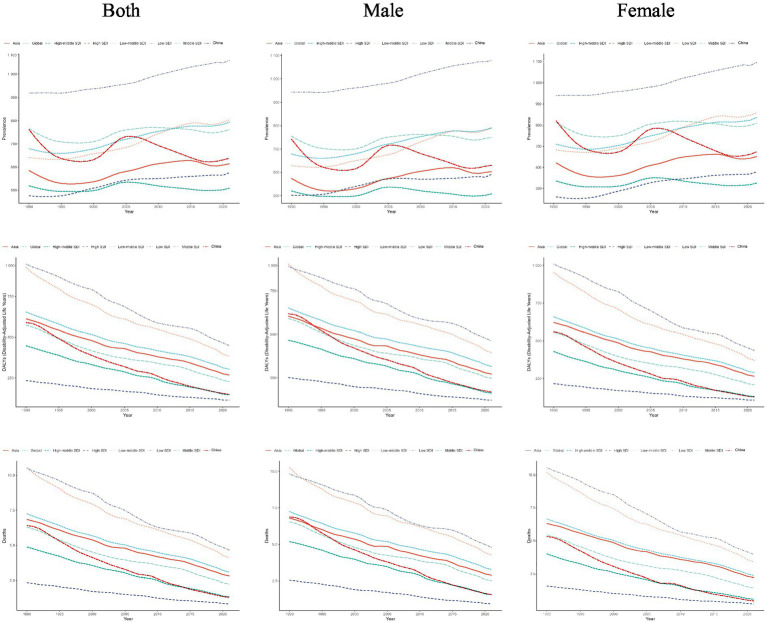
Temporal trends in prevalence, DALYs, and mortality rate of CVDs in children and adolescents by sex and SDI from 1990 to 2021.

Figure 3 revealed that there was a significant declining trend as for DALYs, mortality, across the time, while prevalence presented a gradual upward trend since 2016. These indicators were strongly associated with SDI levels, that is, the lower SDI groups consistently exhibited the higher values, indicating the more severe disease burden. At the global, Asian, and China levels, all the values generally ranged among low-middle and middle SDI regions. In 2021, globally, the prevalence rate of CVDs remained higher in females compared with males (797.060 vs. 791.140 per 100,000). Similarly, the prevalence was higher in females in Asia (625.720 vs. 603.310), also similar pattern in China (646.200 vs. 630.230), per 100,000 population. The prevalence in the global presented the highest upward trend, females: (0.509, 95%CI: 0.417–0.601), males: (0.498, 95%CI: 0.446–0.550), whereas the YLLs in China had the largest downward trend females: (−4.597, 95%CI: −4.767 to −4.427), males: (−4.812, 95%CI: −4.982 to −4.642) (Table 2).

**Table 2:** Mean annual percentage changes, rate in incidence, prevalence, death, DALYs, YLLs, and YLDs due to premature death in China, Asia and the world population under 20 years of age, 1990–2021.

Measures	Male				Female			
	1990	2021	AAPC 95%CI (%)	*p*	1990	2021	AAPC 95%CI (%)	*p*
Global								
Incidence	91.462 (71.583, 115.556)	99.522 (75.873, 129.457)	0.278 (0.232, 0.328)	<0.001	97.719 (76.718, 125.320)	108.559 (81.789, 143.809)	0.341 (0.303, 0.385)	<0.001
Prevalence	678.511 (550.493, 821.914)	791.152 (632.033, 971.938)	0.499 (0.455, 0.550)	<0.001	680.435 (552.540, 826.638)	797.072 (634.283, 985.584)	0.518 (0.422, 0.601)	<0.001
DALYs	651.022 (586.975, 732.696)	315.786 (277.038, 359.558)	−2.291 (−2.352, −2.243)	<0.001	659.003 (583.072, 783.371)	289.716 (256.688, 326.77)	−2.633 (−2.684, −2.595)	<0.001
YLDs	47.717 (33.789, 66.062)	51.492 (34.711, 72.746)	0.256 (0.238, 0.276)	<0.001	52.791 (37.472, 71.963)	55.462 (38.803, 77.043)	0.162 (0.137, 0.197)	<0.001
Deaths	7.241 (6.473, 8.242)	3.275 (2.828, 3.679)	−2.465 (−2.518, −2.429)	<0.001	7.269 (6.397, 8.721)	2.905 (2.545, 3.256)	−2.852(−2.938, −2.782)	<0.001
YLLs	603.311 (536.545, 686.187)	264.291 (227.073, 298.978)	−2.552 (−2.597, −2.518)	<0.001	606.21 (531.71, 732.09)	234.247 (204.359, 263.807)	−2.951(−3.026, −2.889)	<0.001
Asia								
Incidence	87.415 (68.647, 111.278)	81.471 (61.718, 105.739)	−0.230 (−0.297, −0.168)	<0.001	94.573 (74.124, 120.905)	91.585 (69.553, 121.442)	−0.193 (−0.180, −0.018)	<0.001
Prevalence	573.952 (453.031, 700.632)	603.321 (482.077, 741.539)	0.176 (0.058, 0.293)	0.005	597.766 (477.448, 738.479)	625.733 (496.075, 777.166)	0.153 (−0.010, 0.317)	0.0630
DALYs	603.815 (546.637, 676.919)	271.635 (242.742, 303.898)	−2.562 (−2.666, −2.462)	<0.001	621.110 (543.81, 740.483)	265.577 (236.918, 296.509)	−2.762 (−2.854, −2.688)	<0.001
YLDs	42.692 (29.945, 58.978)	41.923 (28.648, 58.580)	−0.052 (−0.115, −0.016)	0.030	50.152 (35.874, 67.811)	47.630 (33.942, 65.006)	−0.162 (−0.226, −0.109)	<0.001
Deaths	6.803 (6.115, 7.677)	2.882 (2.586, 3.209)	−2.718 (−2.819, −2.610)	<0.001	6.908 (6.029, 8.310)	2.741 (2.423, 3.068)	−2.972 (−3.163, −2.787)	<0.001
YLLs	561.125 (502.907, 633.938)	229.712 (204.066, 256.089)	−2.86 (−2.975, −2.766)	<0.001	570.971 (496.773, 690.708)	217.948 (191.412, 244.469)	−3.102 (−3.245, −2.958)	<0.001
China								
Incidence	120.229 (94.720, 154.218)	91.734 (70.732, 116.351)	−0.865 (−0.957, −0.785)	<0.001	123.572 (97.436, 157.598)	95.002 (73.615, 120.769)	−0.843(−0.956, −0.735)	<0.001
Prevalence	742.442 (579.361, 932.333)	630.232 (497.688, 792.819)	−0.537 (−0.619, −0.456)	<0.001	782.529 (614.030, 982.482)	646.207 (516.326, 807.053)	−0.622 (−0.738, −0.516)	<0.001
DALYs	617.658 (541.611, 695.269)	167.222 (140.958, 197.121)	−4.162 (−4.275, −4.047)	<0.001	560.183 (489.926, 639.618)	127.662 (105.587, 148.629)	−4.737 (−4.993, −4.467)	<0.001
YLDs	54.902 (37.898, 76.220)	44.362 (30.366, 61.839)	−0.698 (−0.775, −0.617)	<0.001	67.516 (47.629, 91.836)	52.496 (37.252, 71.826)	−0.829 (−0.902, −0.756)	<0.001
Deaths	6.871 (6.018, 7.892)	1.572 (1.305, 1.868)	−3.512 (−3.643, −3.387)	<0.001	5.902 (5.124, 6.788)	0.947 (0.788, 1.119)	−4.559 (−4.722, −4.388)	<0.001
YLLs	562.757 (490.454, 645.666)	122.868 (101.463, 144.552)	−4.817 (−4.988, −4.641)	<0.001	492.679 (425.376, 568.739)	75.173 (61.887, 88.828)	−4.609 (−4.772, −4.436)	<0.001

### 3.3 Subtypes-specific CVDs metrics in children and adolescents

**Figure 4:**
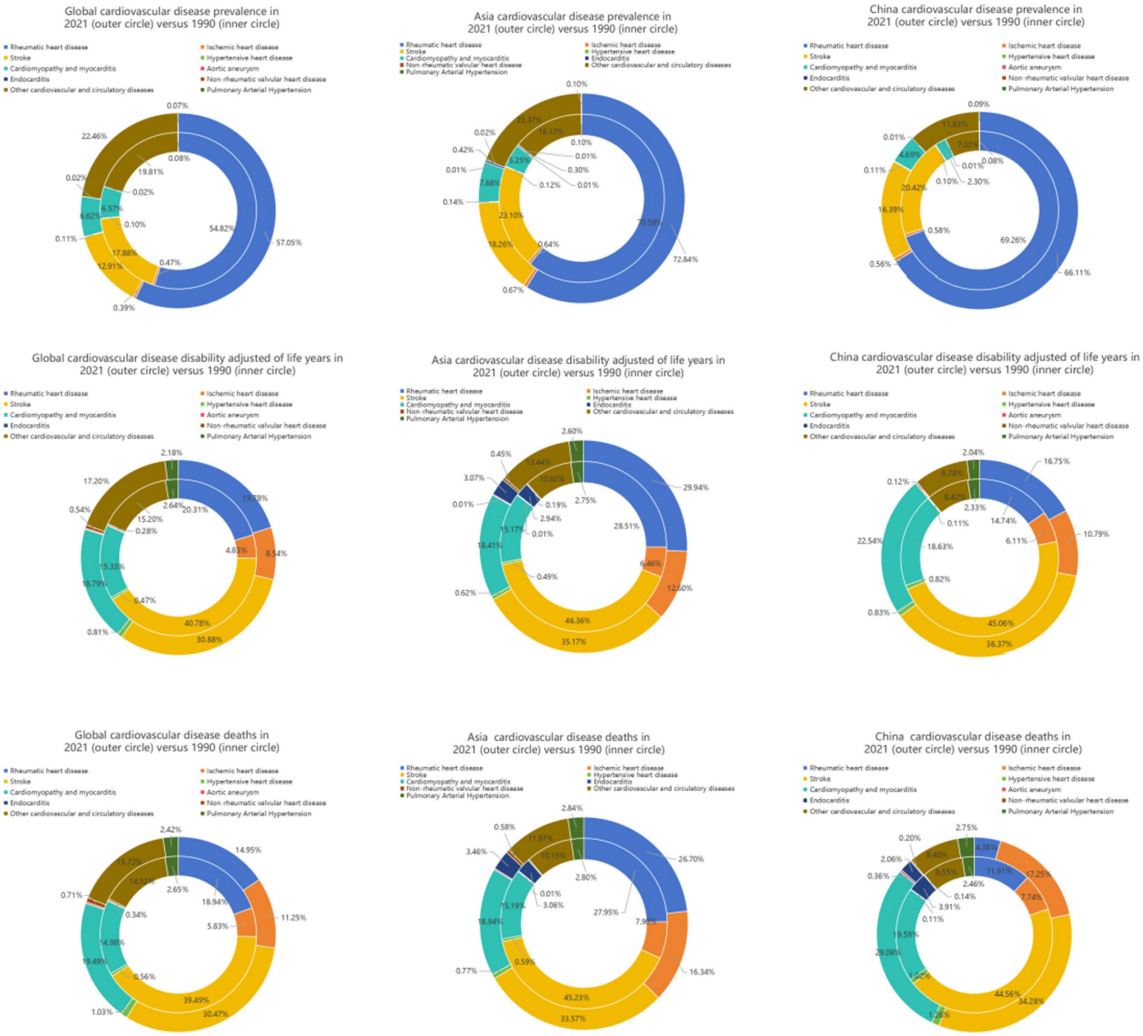
Contribution of prevalence, DALYs, and mortality in China, the Asia, and the world from 1990 to 2021.

Figure 4 presented a comparative analysis of CVDs subtypes among children and adolescents across the global, Asian, and Chinese populations. In terms of global, Asian, and Chinese prevalence indicators, rheumatic heart disease (RHD), other cardiovascular/circulatory diseases, stroke, and cardiomyopathy and myocarditis ranked as the top four position, obviously exceeding 5% proportions. Notably, RHD remained the leading contributor in both 1990 and 2021, accounting for over 50% of prevalent cases. Regarding DALYs, the top four conditions were stroke, cardiomyopathy and myocarditis, non-rheumatic valvular heart disease, and RHD at the three levels, respectively. While stroke remained the primary DALYs burden in both 1990 and 2021, its proportional contribution gradually declined over time. The other three conditions, however, exhibited continuous increases.

In mortality metrics, the top four diseases are stroke, other cardiovascular/circulatory diseases, cardiomyopathy/myocarditis, and RHD across three levels. Similar to the DALYs indicators, stroke continued to serve as the leading mortality factor demonstrating a distinct decline (by at least more than 10%) in 2021 compared to 2019. Interestingly, despite ischemic heart disease was comparatively lower in 1990, it displayed the most significant increase among all the diseases.

### 3.4 Risk factors of children and adolescents CVDs

As evidenced by DALYs, the attributed risk factors for age-specific CVD included high systolic blood pressure, alcohol use, non-optimal ambient temperatures, and metabolic/behavioral/environmental risks. From the global perspective, non-optimal temperatures, hypertension, and metabolic risks served as the primary diseases.

### 3.5 Predictions of children and adolescents CVDs to 2036

**Figure 5:**
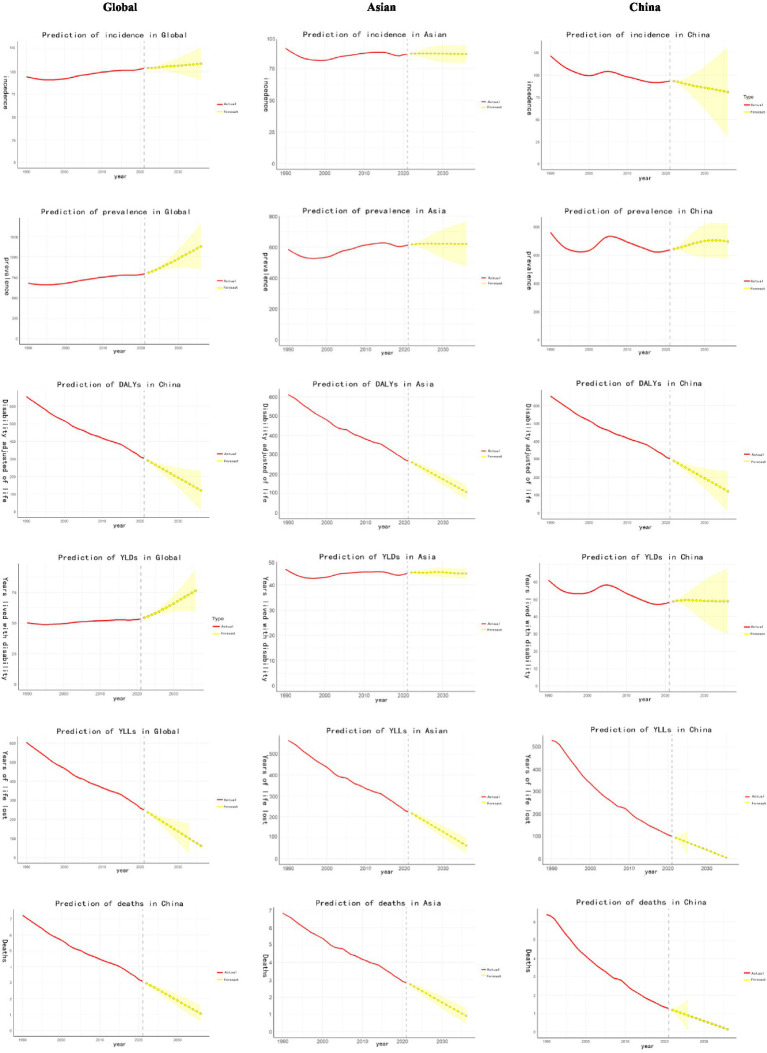
Predicted trends in the burden of CVDs among children and adolescents up to 2036.

Figure 5 showed the long-term trends DALYs for CVDs among individuals under 20 ages from 1990 to 2036. At the global and Asia levels, the incidence and prevalence exhibited the gradual rising trend. For China, the incidence presented a gradual decreasing trend, while the rising trend existed for prevalence. Other indicators, such as DALYs, mortality, and YLLs presented a sharp decreased trend except for YLDs in both Asia and China.

## 4 Discussion

Our study used the best available and robust estimates from the GBD 2021 to explicitly report CVDs epidemics and burden in children and adolescents. In the past three decades, CVDs burden still presented a substantial decline across the world, but it is projected to increase to 2036. Also, a critical public health paradox existed: fatal metrics (mortality and YLLs) have declined significantly, while non-fatal metrics (prevalence and YLDs) have risen substantially. This divergence underscores an ongoing epidemiological transition in pediatric CVDs, that is, from acute and fatal manifestations toward chronic and disabling conditions requiring long-term attention and management.

In terms of births number, China and some Asian countries are experiencing the most significant declining trend (23). This demographic shift may, to some extent, influence the evaluation of the disease epidemic. However, some indicators, including mortality and DALYs, presented the downward trends, aligning with advancements in pediatric healthcare and precision prevention worldwide. The results highlight the urgent need for better understanding of the mediators of cardiometabolic dysfunction in youths for prevention strategies of CVDs in early life. Moreover, the incidence, prevalence and YLDs indicators were higher for females than males, while the other three indicators (mortality, YLLs, and DALYs) showed the opposite direction worldwide. Females also presented a more pronounced decline in AAPC for mortality, YLLs, and DALYs compared to male counterparts. These disparities could be attributable to the combination of physiological properties, daily lifestyle and healthcare-seeking behaviors (24). Those social factors, such as military service participation and sports engagement for males, even enlarge the disparities in CVDs manifestations between genders. Consideration of gender differences is important for prevention, diagnosis, treatment and management of CVDs even for youngsters. The observed characteristics of the epidemics in different regions are of long-term importance, not only in guiding current national policies and strategies in CVDs prevention, but also in predicting future challenges.

In our study, negative associations between SDI and key metrics (DALYs, mortality, and YLLs) for children and adolescents reinforce the evidence that links socioeconomic status with CVDs epidemics, which highlights the imperative of taking actions to address the unique needs of this vulnerable population. Previous studies suggest that low-SDI regions exhibit disproportionately contributions to CVDs mortality from household air pollution (18.480% vs. 0.090%) and lead exposure (2.570% vs. 0.360%) compared with high-SDI regions (4, 19). Targeted interventions for disadvantaged populations through enhancing maternal health and early time nutritional state are essential to prevent CVDs for kids (25–27). Also, RHD and stroke have remained the key contributions to CVDs burden in this young population. Meanwhile, high systolic blood pressure and non-optimal temperature have emerged as critical risk factors for pediatric CVDs (1, 7). Future researches are needed to prioritize accessibility and equality of health workers and parental practices to promote health dynamics.

The global epidemic of childhood obesity and its precipitous upsurge have led to the emergence of CVDs in youngsters, including “adult-onset” diseases at an early age (28). Notably, most children are born with Ideal Cardiovascular Health (ICVH) with specific genetic conditions. The loss of ICVH is a gradual and continuous process, often preceded by prolonged, asymptomatic periods characterized by subclinical changes that eventually manifest as CVDs (29). Approximately 80% of overweight children aged 10–15 develop obesity as approaching adults, perpetuating cardiometabolic risks across the lifespan. Maternal health during lactation and early-life nutritional intake shape the trajectories of CVDs development (30). Early-life interventions, including maternal nutrition optimization and prenatal screening for fetal CVDs risks could prevent the development of CVDs (16). Such measures are vital to further reduce the disease burden in low-resource settings, where disparities in healthcare access exacerbate the burden of non-fatal outcomes. The medical advancements in treating CVDs have not been paralleled in adolescents and young adults, highlighting an urgent need to enhance the management of CVDs in this population, particularly in low-income countries. As for absolute case numbers trajectories of the CVDs epidemiologic transition, the future pattern remain stable, while prediction model to 2036 for the prevalence presents the declining trend as expected. The pathogenesis of CVDs originating in childhood could progressively worsen without timely interventions. Most importantly, monitoring the CVDs burden and promoting the progress in the availability of effective and safe prevention strategies worldwide are really important at early time.

However, this study has several limitations need to be considered. First, as civil registration systems serve as primary sources for mortality statistics, population coverage remains insufficient, especially in those low and middle-income countries. Incomplete medically reporting systems may compromise the quality and the representativeness of CVDs-related mortality data. Second, as children and adolescents CVDs exhibited complex factors with psychological adversity and nutritional factors, these dimensions could not be systematically evaluated due to the unavailability of the data. Moreover, the original data sources from specific countries and periods might influence the accuracy of the GBD estimates and account for the quality of the predictive models (19, 22, 31, 32). Countries and regions with scarce input data should establish high quality databases to help conduct more comprehensive and rigorous research.

In summary, the CVDs burden among children and adolescents is the continuously decreasing feature across the world during the last three decades and continues to rise up to 2036. The burden of overall and type-specific CVDs varied by age, sex, SDI, region, and country, and the marked geographic differences in CVDs epidemics are due to the combined effects of age and other determinants. Given the necessity of devising timely strategies to prevent and control CVDs, concerted efforts of the health workforce in the targeted implementation of effective primary prevention strategies to mitigate the disease burden.

## Data Availability

The original contributions presented in the study are included in the article/Supplementary material, further inquiries can be directed to the corresponding authors.

## Footnotes

1
http://ghdx.healthdata.org
