# Supplementary material for: Burden and trend of cardiovascular diseases in youths aged 0–19 years in China, Asia, and the world, with forecasts to 2036: a systematic analysis of the global burden of disease study 2021
Source: Front Public Health. 2026 Jan 23;13:1653981. doi: 10.3389/fpubh.2025.1653981 (PMC12880115; doi:10.3389/fpubh.2025.1653981)
Supplement: Supplementary file 1 [file Table_1.docx]

| **Supplementary Table1a.** Mean annual percentage changes, rate in age-group incidence, prevalence, death, DALYs, YLLs, and YLDs of CVDs in **global** among children and adolescents, 1990-2021 | | | | |
| --- | --- | --- | --- | --- |
| metric | 1990(rate) | 2021 | AAPC 95%CI (%) | *P* |
| **<5 years** | | | | |
| Incidence | 61.466(50.100,75.816) | 58.090(45.989,73.92) | -0.181(-0.240,-0.121) | <0.001 |
| Prevalence | 242.532(204.408,288.273) | 287.023(235.376,348.756) | 0.544(0.519,0.569) | <0.001 |
| DALYs | 1362.089(1188.346,1647.133) | 469.567(383.946,562.196) | -3.412(-3.524,-3.301) | <0.001 |
| YLDs | 20.102(13.835,27.752) | 21.782(15.022,30.399) | 0.256(0.235,0.276) | <0.001 |
| Deaths | 15.118(13.092,18.328) | 5.039(4.055,6.093) | -3.526(-3.626,-3.425) | <0.001 |
| YLLs | 1341.987(1162.769,1625.92) | 447.785(360.593,541.296) | -3.522(-3.622,-3.422) | <0.001 |
| **5-9 years** | | | | |
| Incidence | 89.082(61.66,125.591) | 96.250(64.004,140.975) | 0.255(0.170,0.340) | <0.001 |
| Prevalence | 543.190(440.018,660.104) | 611.981(484.703,753.482) | 0.384(0.321,0.446) | <0.001 |
| DALYs | 290.720(253.62,335.676) | 140.260(121.776,163.542) | -2.300(-2.532,-2.068) | <0.001 |
| YLDs | 41.613(28.819,58.505) | 42.84(29.177,60.779) | 0.097(0.081,0.112) | <0.001 |
| Deaths | 3.006(2.586,3.505) | 1.176(1.040,1.332) | -2.596(-2.788,-2.404) | <0.001 |
| YLLs | 249.107(214.342,290.516) | 97.420(86.103,110.329) | -2.597(-2.789,-2.405) | <0.001 |
| **10-14 years** | | | | |
| Incidence | 109.256(69.427,163.356) | 122.716(74.379,188.761) | 0.377(0.335,0.418) | <0.001 |
| Prevalence | 864.346(659.613,1111.32) | 989.62(743.011,1292.828) | 0.441(0.357,0.524) | <0.001 |
| DALYs | 284.443(255.132,320.665) | 186.883(163.067,217.54) | -1.353(-1.570,-1.135) | <0.001 |
| YLDs | 62.032(41.545,86.067) | 64.99(43.435,92.67) | 0.156(0.119,0.193) | <0.001 |
| Deaths | 2.868(2.581,3.237) | 1.573(1.425,1.74) | -1.696(-1.930,-1.461) | <0.001 |
| YLLs | 222.411(200.173,251.053) | 121.893(110.453,134.842) | -1.697(-1.933,-1.461) | <0.001 |
| **15-19 years** | | | | |
| Incidence | 124.814(86.094,176.325) | 140.525(93.884,204.328) | 0.383(0.368,0.397) | <0.001 |
| Prevalence | 1163.296(883.526,1520.307) | 1320.248(978.494,1762.136) | 0.406(0.354,0.458) | <0.001 |
| DALYs | 602.091(561.025,655.078) | 431.157(391.040,481.903) | -1.113(-1.347,-0.878) | <0.001 |
| YLDs | 83.510(57.304,119.006) | 86.055(56.621,125.919) | 0.094(0.065,0.124) | <0.001 |
| Deaths | 7.148(6.713,7.657) | 4.759(4.380,5.175) | -1.300(-1.441,-1.157) | <0.001 |
| YLLs | 518.580(487.017,555.500) | 345.102(317.602,375.356) | -1.304(-1.445,-1.162) | <0.001 |

| **Supplementary Table1b.** Mean annual percentage changes, rate in age-group incidence, prevalence, death, DALYs, YLLs, and YLDs of CVDs in **Asia** among children and adolescents, 1990-2021 | | | | |
| --- | --- | --- | --- | --- |
| metric | 1990(rate) | 2021 | AAPC 95%CI (%) | *P* |
| **<5 years** | | | | |
| Incidence | 58.929(47.98,72.618) | 46.351(36.059,59.453) | -0.773(-0.896,-0.650) | <0.001 |
| Prevalence | 161.113(137.187,189.979) | 178.667(149.862,216.618) | 0.336(0.301,0.370) | <0.001 |
| DALYs | 1184.429(1033.054,1416.957) | 358.681(293.78,427.318) | -3.823(-3.979,-3.666) | <0.001 |
| YLDs | 15.164(10.467,20.622) | 15.316(10.407,21.167) | 0.021(-0.031,0.072) | 0.429 |
| Deaths | 13.175(11.429,15.8) | 3.859(3.126,4.624) | -3.932(-4.091,-3.773) | <0.001 |
| YLLs | 1169.265(1015.264,1401.672) | 343.365(278.179,411.153) | -3.925(-4.084,-3.765) | <0.001 |
| **5-9 years** | | | | |
| Incidence | 83.856(58.766,116.835) | 76.401(51.891,109.406) | -0.287(-0.356,-0.217) | <0.001 |
| Prevalence | 444.327(359.472,547.063) | 459.865(365.977,569.582) | 0.116(0.048,0.183) | 0.001 |
| DALYs | 288.908(245.021,338.742) | 121.762(106.621,140.033) | -2.797(-3.242,-2.351) | <0.001 |
| YLDs | 36.875(25.847,51.627) | 34.923(24.304,49.087) | -0.171(-0.195,-0.147) | <0.001 |
| Deaths | 3.041(2.55,3.627) | 1.049(0.931,1.186) | -3.441(-4.046,-2.832) | <0.001 |
| YLLs | 252.033(211.338,300.584) | 86.839(77.086,98.189) | -3.443(-4.049,-2.834) | <0.001 |
| **10-14 years** | | | | |
| Incidence | 123.471(85.236,173.582) | 98.036(60.506,148.006) | -0.140(-0.263,-0.017) | 0.026 |
| Prevalence | 1062.133(791.952,1406.982) | 767.64(570.045,996.973) | 0.084(-0.007,0.174) | 0.069 |
| DALYs | 644.142(594.369,703.383) | 174.298(152.58,199.187) | -1.563(-1.759,-1.366) | <0.001 |
| YLDs | 80.961(55.536,114.395) | 54.3(36.526,76.094) | -0.188(-0.247,-0.129) | <0.001 |
| Deaths | 7.764(7.192,8.408) | 1.549(1.384,1.724) | -1.989(-2.185,-1.793) | <0.001 |
| YLLs | 563.181(521.697,609.855) | 119.998(107.228,133.594) | -1.991(-2.188,-1.794) | <0.001 |
| **15-19 years** | | | | |
| Incidence | 123.471(85.236,173.582) | 123.209(83.783,174.475) | -0.011(-0.030,0.007) | 0.240 |
| Prevalence | 1062.133(791.952,1406.982) | 1038.332(770.192,1385.147) | -0.072(-0.203,0.058) | 0.277 |
| DALYs | 644.142(594.369,703.383) | 437.38(398.495,480.998) | -1.254(-1.703,-0.803) | <0.001 |
| YLDs | 80.961(55.536,114.395) | 73.276(49.316,105.782) | -0.322(-0.393,-0.250) | <0.001 |
| Deaths | 7.764(7.192,8.408) | 5.022(4.608,5.467) | -1.413(-1.964,-0.859) | <0.001 |
| YLLs | 563.181(521.697,609.855) | 364.104(334.137,396.441) | -1.414(-1.971,-0.854) | <0.001 |

| **Supplementary Table1c.**Mean annual percentage changes,rate in age-group incidence, prevalence, death, DALYs, YLLs, and YLDs of CVDs in **China** among children and adolescents, 1990-2021 | | | | |
| --- | --- | --- | --- | --- |
| metric | 1990(rate) | 2021 | AAPC 95%CI (%) | P |
| **<5 years** | | | | |
| Incidence | 93.728(78.503,112.376) | 61.609(48.681,78.715) | -1.356(-1.441,-1.271) | <0.001 |
| Prevalence | 162.800(138.156,192.941) | 151.541(126.069,181.248) | -0.226(-0.352,-0.099) | 0.001 |
| DALYs | 1236.893(1051.983,1459.122) | 140.451(113.37,168.844) | -6.848(-7.162,-6.532) | <0.001 |
| YLDs | 17.02(12.022,22.587) | 13.809(9.45,18.659) | -0.671(-0.786,-0.557) | <0.001 |
| Deaths | 13.743(11.676,16.279) | 1.4220(1.121,1.761) | -5.732(-6.001,-5.463) | <0.001 |
| YLLs | 1219.872(1036.332,1444.431) | 126.642(99.854,156.69) | -5.722(-5.968,-5.476) | <0.001 |
| **5-9 years** | | | | |
| Incidence | 123.555(86.885,169.862) | 92.496(63.067,130.467) | -0.927(-1.004,-0.850) | <0.001 |
| Prevalence | 535.413(429.951,664.996) | 468.501(374.524,579.243) | -0.402(-0.448,-0.356) | <0.001 |
| DALYs | 258.091(229.77,289.8) | 84.294(69.006,99.772) | -3.629(-4.163,-3.091) | <0.001 |
| YLDs | 45.835(31.961,63.197) | 36.253(24.919,50.36) | -0.754(-0.778,-0.729) | <0.001 |
| Deaths | 2.560(2.287,2.878) | 0.580(0.482,0.681) | -3.686(-4.085,-3.286) | <0.001 |
| YLLs | 212.255(189.625,238.555) | 48.041(39.918,56.436) | -3.760(-4.218,-3.303) | <0.001 |
| **10-14 years** | | | | |
| Incidence | 136.916(87.61,202.334) | 107.079(66.221,160.484) | -0.794(-0.858,-0.730) | <0.001 |
| Prevalence | 976.854(723.959,1300.153) | 866.512(655.256,1147.473) | -0.364(-0.435,-0.294) | <0.001 |
| DALYs | 238.984(209.028,273.866) | 116.709(93.785,143.345) | -2.361(-2.675,-2.046) | <0.001 |
| YLDs | 75.911(51.17,107.137) | 63.083(42.865,89.049) | -0.588(-0.635,-0.542) | <0.001 |
| Deaths | 2.105(1.888,2.336) | 0.693(0.584,0.802) | -3.685(-4.182,-3.185) | <0.001 |
| YLLs | 163.073(146.236,180.937) | 53.626(45.164,62.059) | -3.178(-3.493,-2.862) | <0.001 |
| **15-19 years** | | | | |
| Incidence | 133.033(88.948,192.334) | 111.181(73.918,159.525) | -0.569(-0.611,-0.528) | <0.001 |
| Prevalence | 1302.836(957.139,1724.454) | 1096.119(805.383,1438.048) | -0.553(-0.610,-0.497) | <0.001 |
| DALYs | 575.904(503.698,644.617) | 277.274(232.311,330.581) | -2.327(-2.493,-2.160) | <0.001 |
| YLDs | 100.112(68.255,143.116) | 81.867(56.169,117) | -0.643(-0.671,-0.615) | <0.001 |
| Deaths | 6.564(5.78,7.431) | 2.697(2.202,3.235) | -2.631(-2.766,-2.495) | <0.001 |
| YLLs | 475.792(418.98,538.704) | 195.406(159.548,234.347) | -2.641(-2.777,-2.504) | <0.001 |
