# Supplementary material for: Burden and trend of cardiovascular diseases in youths aged 0–19 years in China, Asia, and the world, with forecasts to 2036: a systematic analysis of the global burden of disease study 2021
Source: Front Public Health. 2026 Jan 23;13:1653981. doi: 10.3389/fpubh.2025.1653981 (PMC12880115; doi:10.3389/fpubh.2025.1653981)
Supplement: Supplementary file 2 [file Data_Sheet_1.pdf]

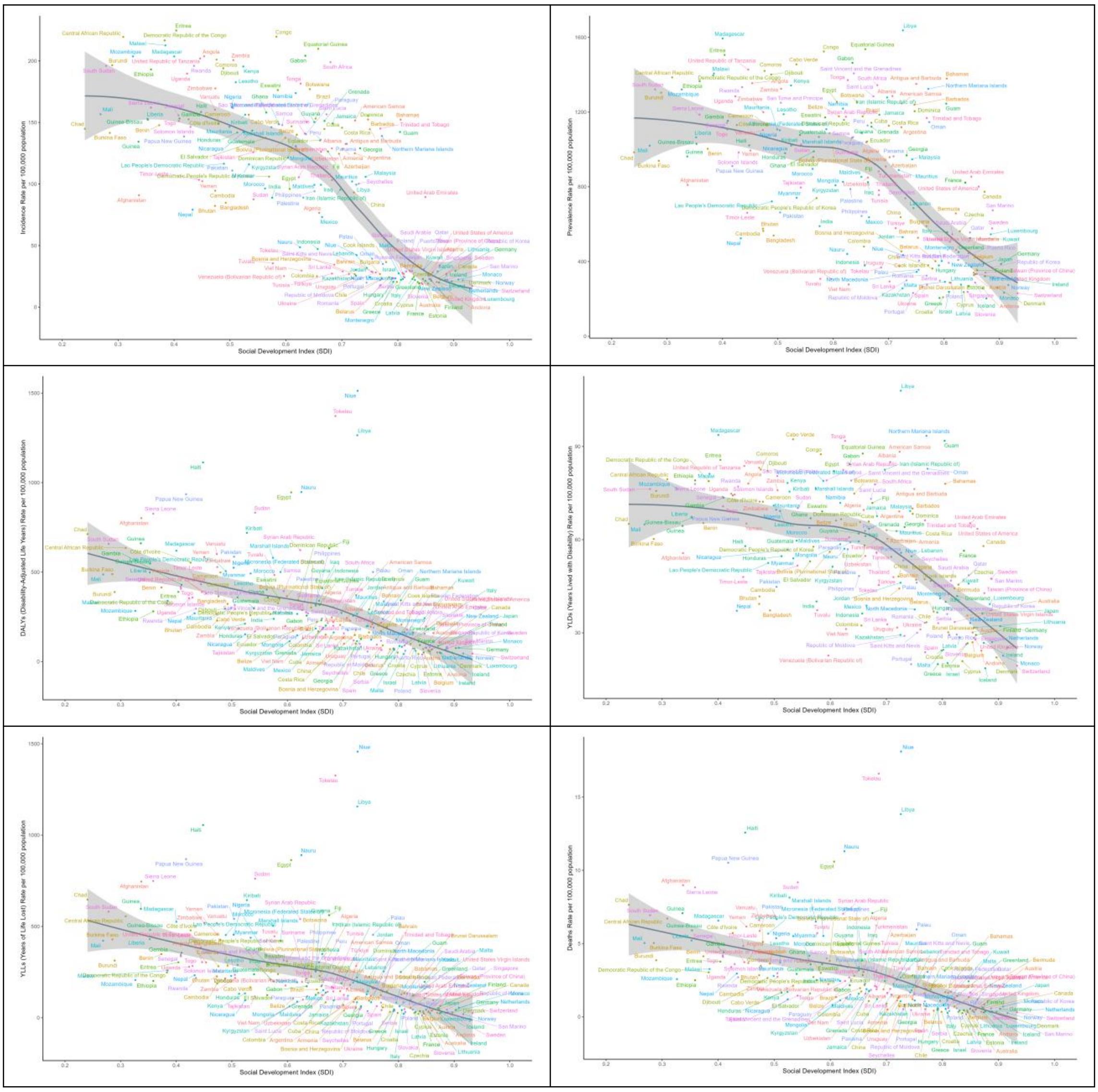

Supplementary Fig1. Incidence, prevalence, DALYs, YLLs, YLDs and mortality for childhood CVDs across 204 Global Burden of Disease (GBD) countries/territories ,stratified by SDI from 1990 to 2021.

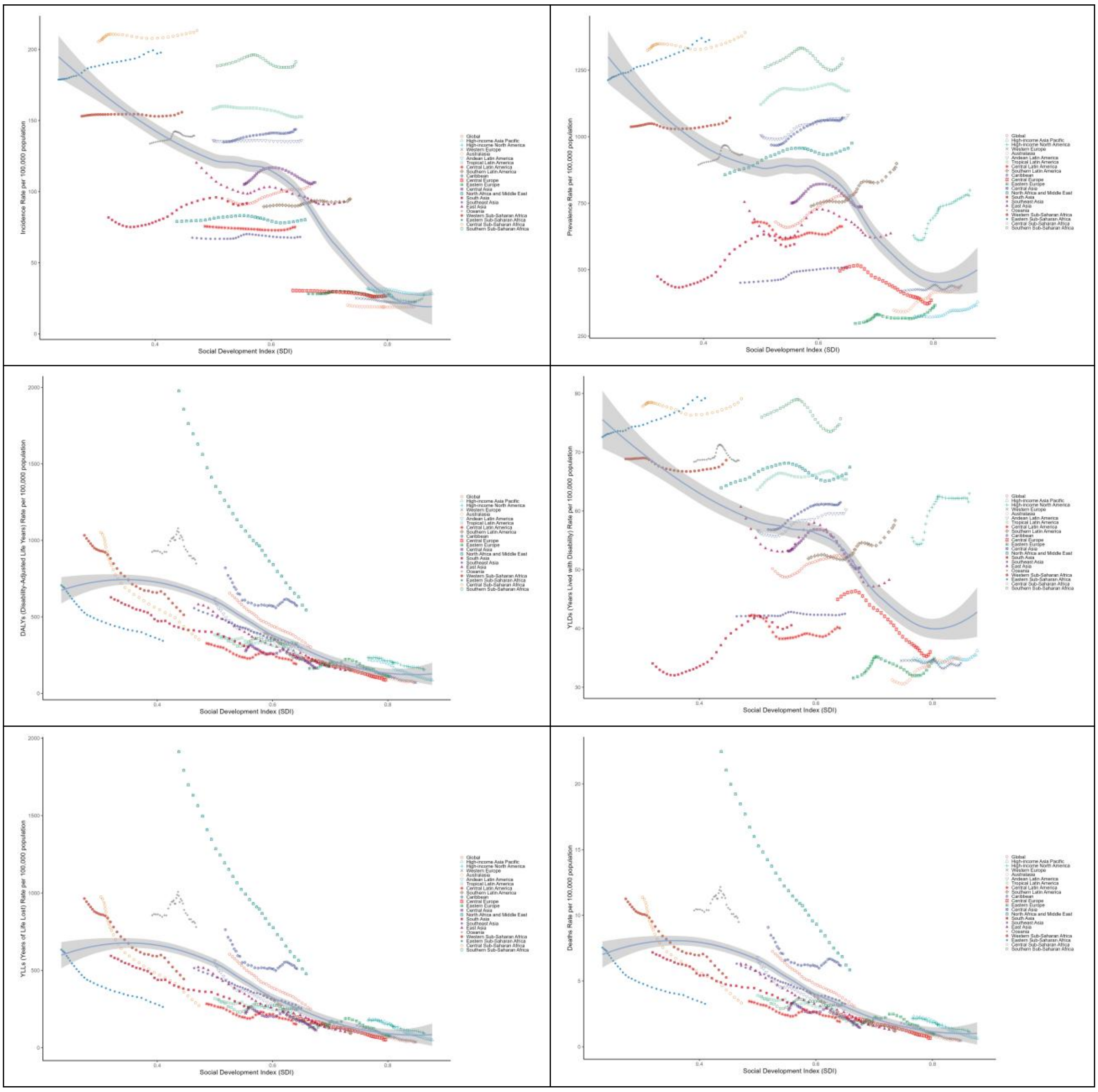

Supplementary Fig2. Incidence, prevalence, DALY, YLLs, YLDs and mortality for childhood CVDs across 21 Global Burden of Disease (GBD) regions , stratified by SDI from 1990 to 2021.
